# Supplementary material for: Microblog topic identification using Linked Open Data
Source: PLoS One. 2020 Aug 11;15(8):e0236863. doi: 10.1371/journal.pone.0236863 (PMC7418982; doi:10.1371/journal.pone.0236863)
Supplement: S1 Appendix — (PDF) [file pone.0236863.s001.pdf]

S1 Appendix The main object properties of topico:Topic

The main object properties for *Topico* are provided. First the syntax is summarized in the following table. Then, the object properties are provided. The properties with inverse properties are indicated within their definition and not further defined in the table.

The basic syntax of OWL-DL.

Descriptions	Abstract Syntax	DL Syntax
Concepts	$intersection(C_1, C_2, \cdots, C_n)$	$C_1 \sqcap C_2 \sqcap \cdots \sqcap C_n$
	$union(C_1, C_2, \cdots, C_n)$	$C_1 \sqcup C_2 \sqcup \cdots \sqcup C_n$
	$partial(C_1, C_2, \cdots, C_n)$	$A \sqsubseteq C_1 \sqcap C_2 \sqcap \cdots C_n$
	$complete(C_1, C_2, \cdots, C_n)$	$A \equiv C_1 \sqcap C_2 \sqcap \cdots C_n$
	universal concept (top)	$\top$
Roles	existential restriction	$\exists R.C$
	universal restriction	$\forall R.C$
	cardinality restriction	$[= \mid \geq \mid \leq] nR.C$
	Inverse of R	R^-

Object Properties	Description Logic
`:hasAgent`	$\sqsubseteq \forall$ `:hasAgent.foaf`:Agent $\sqcap$ $\exists$ `:hasAgent.`$\top \sqsubseteq$:Topic<br>$\equiv$ `:isAnAgentOf`$^-$
`:hasPerson`	$\sqsubseteq$ `:hasAgent`<br>$\exists$ `:hasPerson.`$\top \sqsubseteq$:Topic $\sqcap$ $\top \sqsubseteq \forall$ `:hasPerson.foaf`:Person<br>$\equiv$ `:isAPersonf`$^-$
`:hasGroup`	$\sqsubseteq$ `:hasAgent`<br>$\exists$ `:hasGroup.`$\top \sqsubseteq$:Topic $\sqcap$ $\forall$ `:hasGroup.foaf`:Group<br>$\equiv$ `:isAGroupOf`$^-$
`:hasOrganization`	$\sqsubseteq$ `:hasAgent`<br>$\exists$ `:hasOrganization.`$\top \sqsubseteq$:Topic $\sqcap$ $\forall$ `:hasOrganization.foaf`:Organization<br>$\equiv$ `:isAnOrganizationf`$^-$
`:hasLocation`	$\sqsubseteq \forall$ `:hasLocation.`:Location $\sqcap$ $\exists$ `:hasLocation.`$\top \sqsubseteq$:Topic<br>$\equiv$ `:isLocationOf`$^-$
`:hasTemporalExpression`	$\sqsubseteq \forall$ `:hasTemporalExpression.`:TemporalExpression $\sqcap$

	$\exists$:hasTemporalExpression.$\top \sqsubseteq$:Topic<br>$\equiv$:isTemporalExpressionOf^{-}
:hasTemporalEntity	$\sqsubseteq$:hasTemporalExpression<br>$\forall$:hasTemporalEntity.time:TemporalEntity $\sqcap$<br>$\exists$:hasTemporalEntity.$\top \sqsubseteq$:Topic<br>:isTemporalEntityOf^{-}
:hasTemporalTerm	$\sqsubseteq$:hasTemporalExpression<br>$\forall$:hasTemporalTerm.:TemporalTerm $\sqcap$<br>$\exists$:hasTemporalTerm.$\top \sqsubseteq$:Topic<br>$\equiv$:isTemporalTermOf^{-}
:isAbout	$\sqsubseteq \forall$:isAbout.$\top \sqcap \exists$:isAbout.$\top \sqsubseteq$:Topic<br>$\equiv$:inTopic^{-}
:observationInterval	$\top \sqsubseteq = 1$:observationInterval.$\top \sqcap$<br>$\exists$:observationInterval.$\top \sqsubseteq$:Topic $\sqcap$<br>$\forall$:observationInterval.time:Interval<br>$\equiv$:isTopicOfObservationInterval^{-}
