# Supplementary figures and images for: Microblog topic identification using Linked Open Data
Source: PLoS One. 2020 Aug 11;15(8):e0236863. doi: 10.1371/journal.pone.0236863 (PMC7418982; doi:10.1371/journal.pone.0236863)

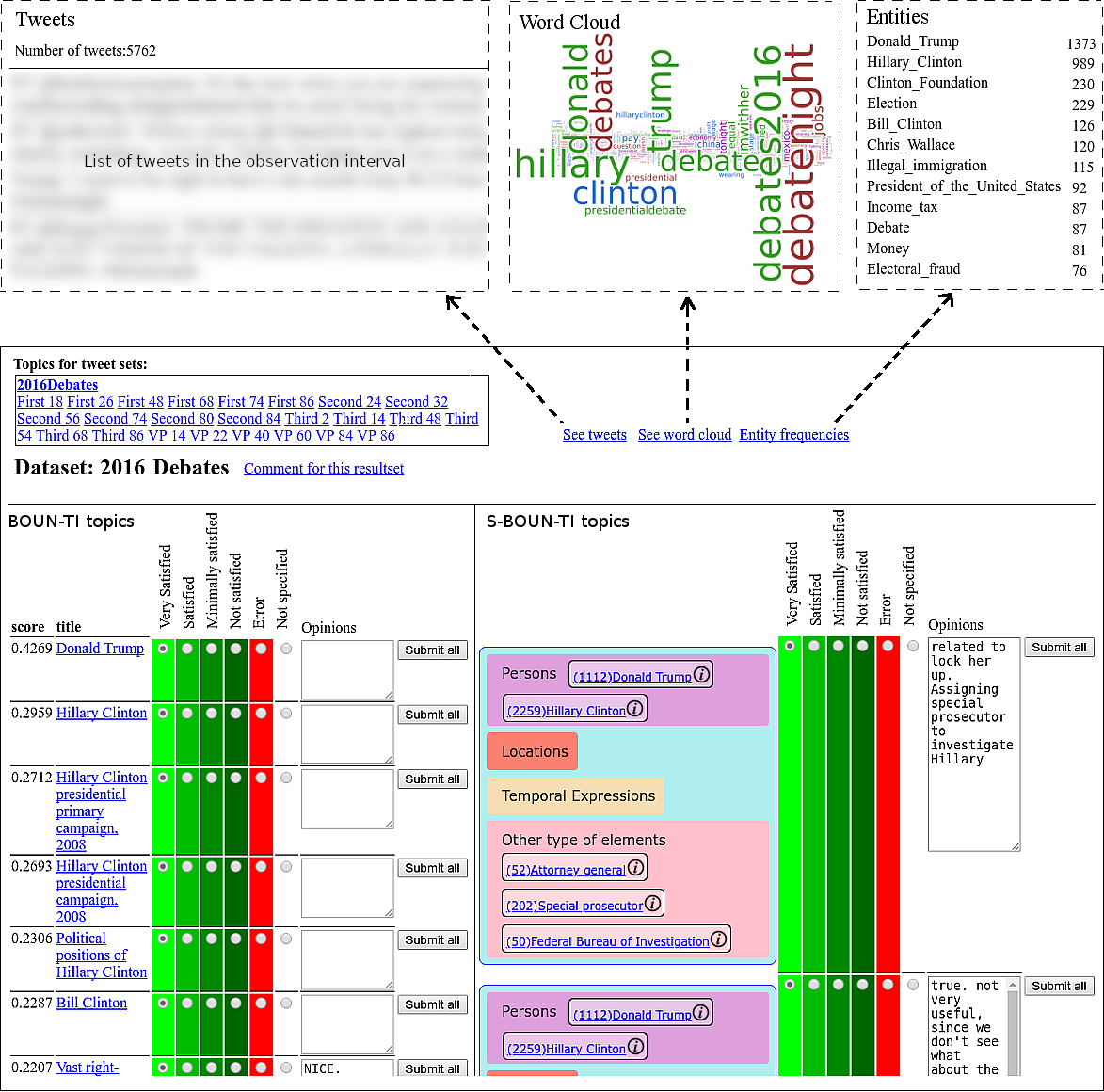

Supplement: S1 Fig — The See tweets and See word cloud links show the related tweets and a word cloud generated from them. The Entity frequencies link shows the list of linked entities and their frequencies. All resources are reachable trough web-links for inspection. (PNG) [file pone.0236863.s002.png]

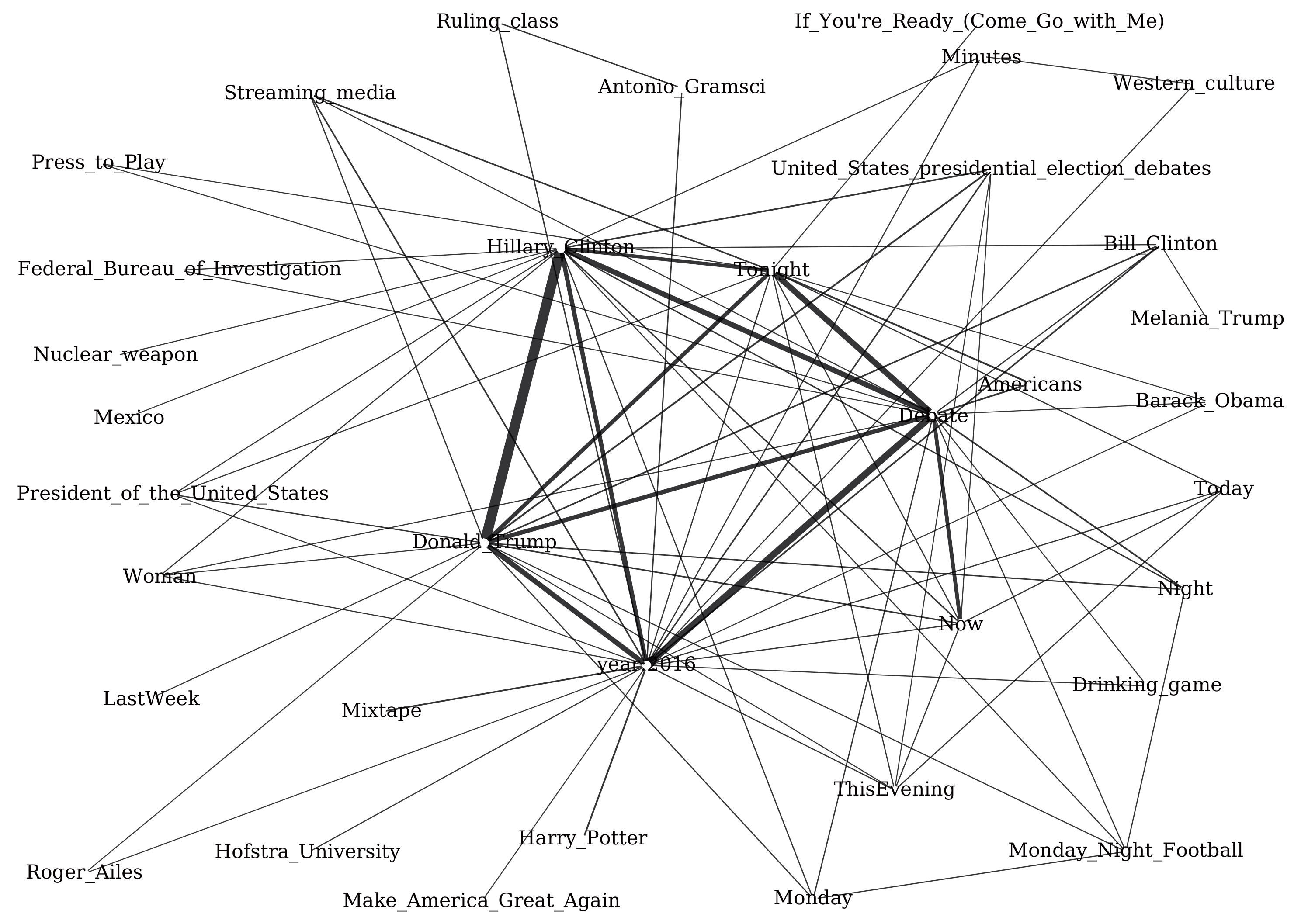

Supplement: S2 Fig — (PNG) [file pone.0236863.s003.png]

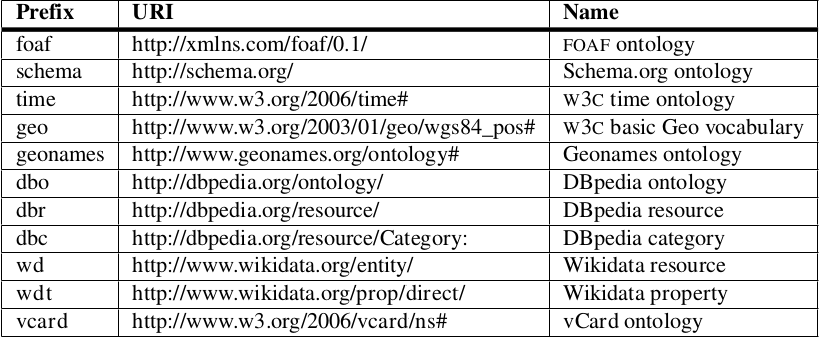

Supplement: S1 Table — (PNG) [file pone.0236863.s004.png]

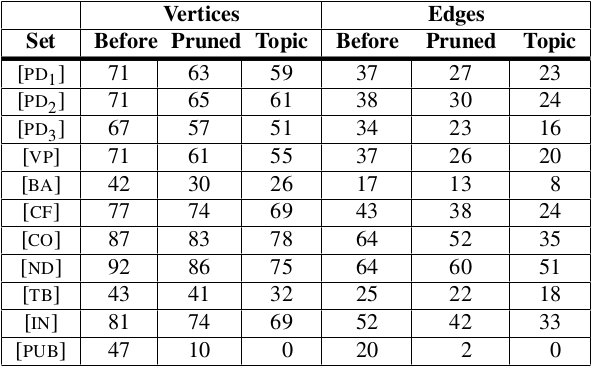

Supplement: S2 Table — This table shows the percentage of tweets in the post sets that produce the vertices (topic elements), edges (co-occurring elements), and topics. The columns labeled Before and Pruned show the impact of pruning the graph. The columns labeled Topic show how many were retained in the topic. (PNG) [file pone.0236863.s005.png]

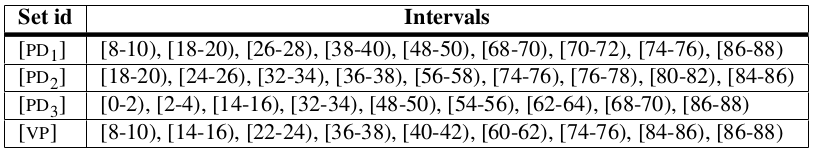

Supplement: S3 Table — (PNG) [file pone.0236863.s006.png]
